# Clear cell sarcoma of the kidney with calcification and a novel chromosomal abnormality: a case report

**DOI:** 10.1186/s13000-015-0352-7

**Published:** 2015-07-17

**Authors:** Masaya Kato, Yuya Sato, Keitaro Fukushima, Mayuko Okuya, Hidemitsu Kurosawa, Shigeko Kuwashima, Koichi Honma, Kentaro Okamoto, Takashi Tsuchioka, Osamu Arisaka

**Affiliations:** Department of Pediatrics, Dokkyo Medical University, 880 Kita-Kobayashi, Mibu, Tochigi 321-0293 Japan; Department of Radiology, Dokkyo Medical University, 880 Kita-Kobayashi, Mibu, Tochigi 321-0293 Japan; Department of Anatomic and Diagnostic Pathology, Dokkyo Medical University, 880 Kita-Kobayashi, Mibu, Tochigi 321-0293 Japan; First Department of Surgery, Dokkyo Medical University, 880 Kita-Kobayashi, Mibu, Tochigi 321-0293 Japan

**Keywords:** Clear cell sarcoma, Kidney, Calcification, Chromosomal abnormality

## Abstract

A 9-year-old male presented with a renal tumor that showed a cystic structure with calcification on computed tomography. A pathological analysis of the resected tumor suggested clear cell sarcoma of the kidney (CCSK). Thus, this patient suffered atypical CCSK with significant calcification and gross necrosis. A novel chromosomal abnormality was also identified in the tumor.

## Background

Clear cell sarcoma of the kidney (CCSK) is a rare renal tumor in children, second to Wilms tumor. CCSK represents 2–9 % of all pediatric renal tumors and generally arises before the age of 5 years [1, 2]. The male-to-female ratio is almost 2:1 [1, 2]. Its typical features are a mucoid texture, necrotic foci, and prominent cyst formation. Multiple histological patterns (classic, myxoid, sclerosing, cellular, epithelioid, palisading, spindle, storiform, and anaplastic) are blended together [1]. A CCSK in a 9-year-old male is described with an atypical pathological status, including gross necrosis and calcification.

## Case presentation

**Fig. 1:**
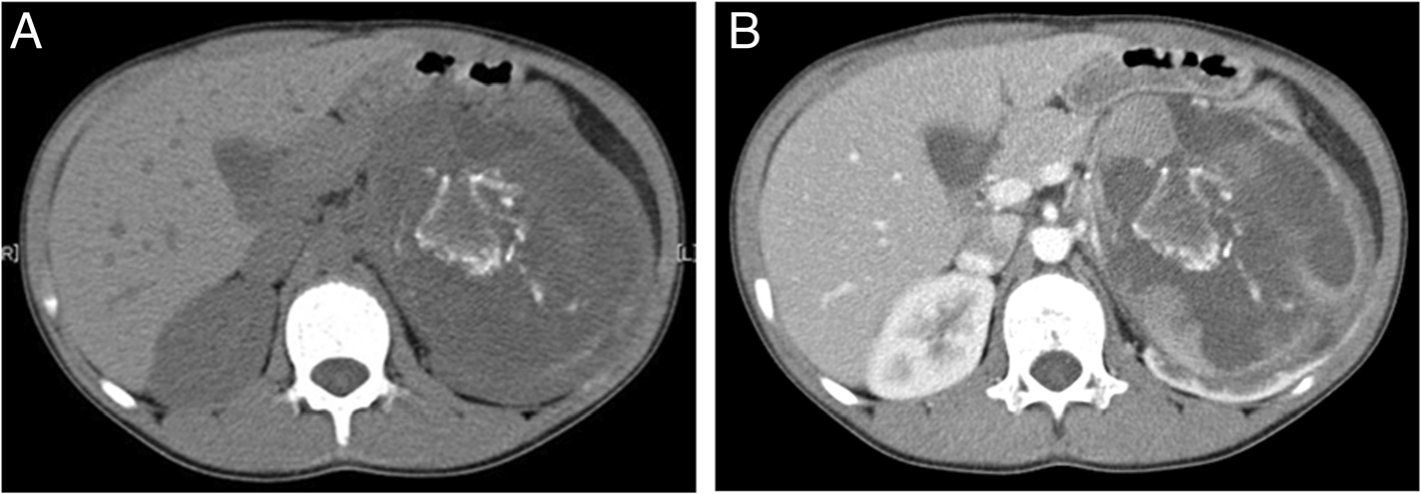
Computed tomography (CT) findings. **a** Uncontrasted CT shows a tumor with calcification in the left kidney. **b** Contrast-enhanced CT shows heterogeneous enhancement and completely enclosed by the renal capsule

**Fig. 2:**
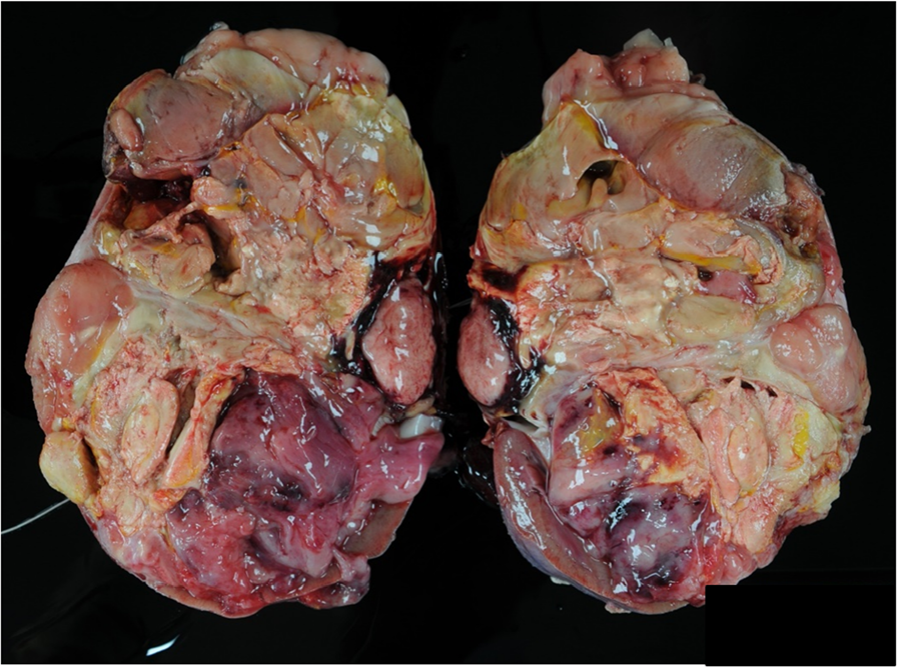
Gross pathological findings. The tumor weighed approximately 750 g and measured 15 × 12 × 9 cm. The left kidney is replaced by the neoplasm. Calcification is apparent around the necrotic tissue

**Fig. 3:**
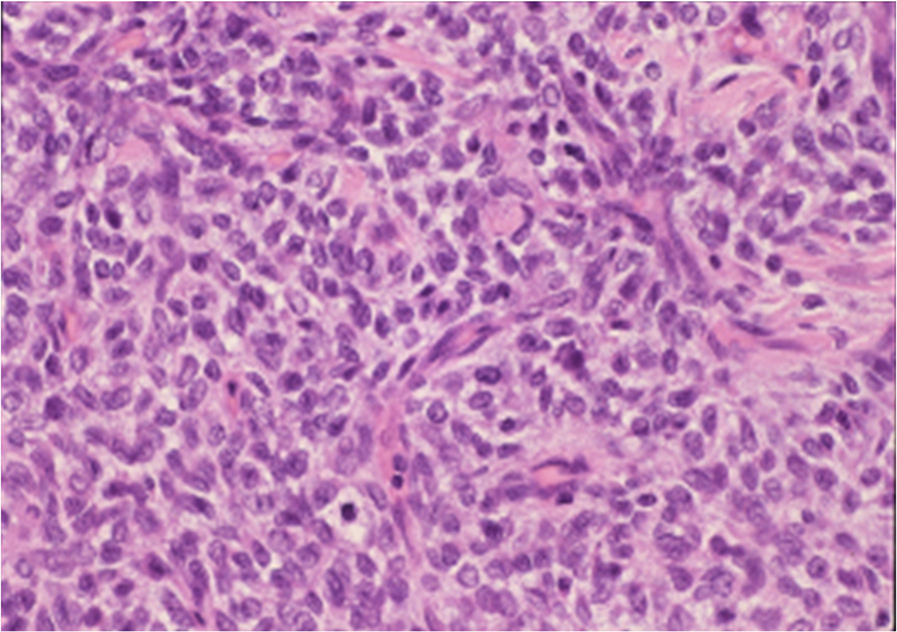
Microscopic pathological analysis. Small oval cells arranged in nests or cords are separated by fibrovascular septa. Plump or spindle-shaped cells contain clear cytoplasm

A 9-year-old boy complained of abdominal bloating. Blood parameters, including neuron specific enolase, alpha fetoprotein, and beta human chorionic gonadotropin, were in the normal ranges, and his urinary vanillylmandelic acid and homovanillic acid were not elevated. Unenhanced abdominal computed tomography (CT) revealed a tumor with calcification in the left kidney, with infiltration of the renal sinus and 15 cm wide (Fig. 1a). Contrast-enhanced CT revealed a heterogeneously enhancing mass completely enclosed by the renal capsule (Fig. 1b). No metastasis was detected in the lungs, liver, or bone by analysis of CT, magnetic resonance imaging and ^99m^Tc scintigraphy. After complete surgical resection, the tumor showed heterogeneous pathological tissues divided by septa and included necrotic tissue (Fig. 2). Calcification was found around the necrotic tissue, but no macroscopic osteoplasty was observed. Pathological analysis suggested a monomorphic malignant neoplasm composed of plump or spindle-shaped cells with elongated, vesicular nuclei or small nuclei and variable amounts of clear cytoplasm (Fig. 3). These results prompted a diagnosis of stage 2 clear cell sarcoma of the kidney (CCSK).

**Fig. 4:**
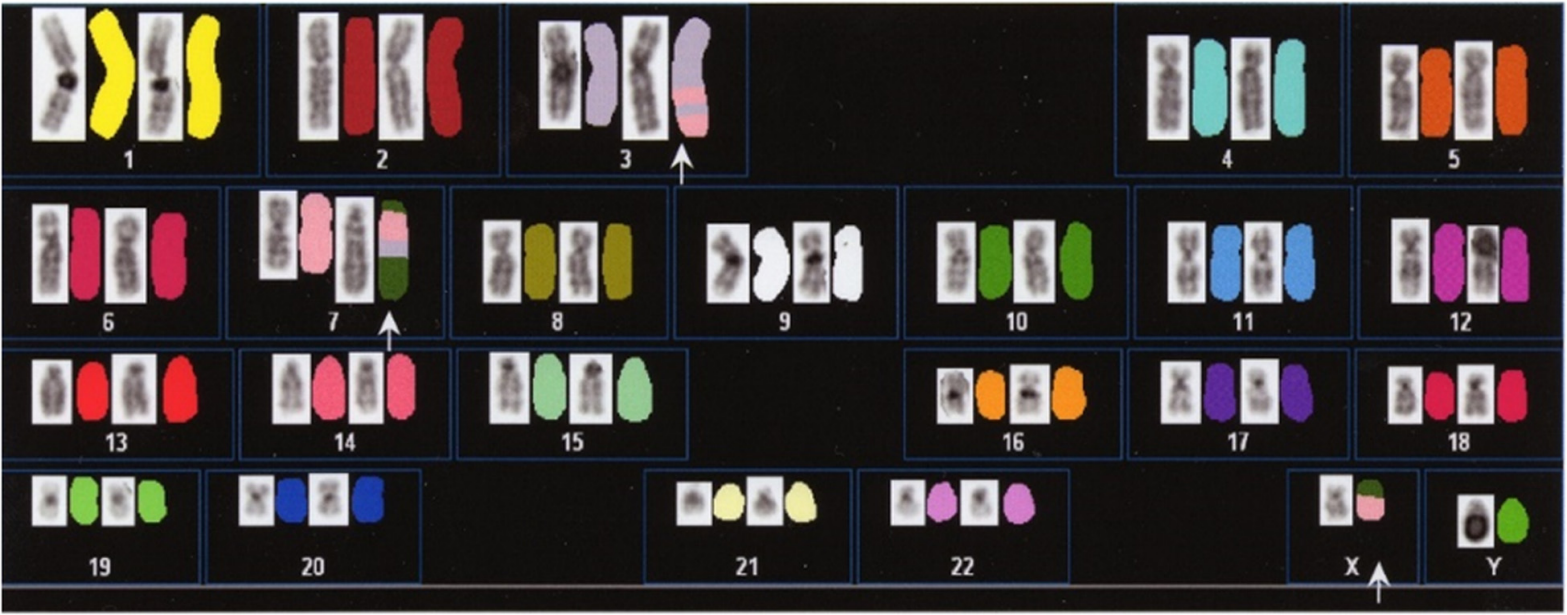
Fluorescence *in situ* hybridization of tumor chromosomes. A new chromosomal abnormality, 46,XY,der(3)(3pter → 3q23::7?::3?::7p13 → 7pter)der(7)(X?::7p11.2 → 7q22::3?::Xq13 → Xqter)der(X)del(X)(p11.2p22.1)t(X;7)(q11;?), was identified in the tumor

The 20 CCSK cells analyzed with fluorescence *in situ* hybridization (FISH) had an abnormal karyotype, 46,XY,der(3)(3pter → 3q23::7?::3?::7p13 → 7pter)der(7)(X?::7p11.2 → 7q22::3?::Xq13 → Xqter)der(X)del(X)(p11.2p22.1)t(X;7)(q11;?) (Fig. 4). This abnormal chromosome has not been reported previously in CCSK.

Chemotherapy was administered according to the Japanese Wilms Tumor Study (JWiTS)-2 Regimen I, including vincristine, doxorubicin, and cyclophosphamide. The patient has remained in complete remission for 3 years.

## Discussion

In terms of its pathological features, CCSK is most commonly described as a tan-grey, soft, and mucoid mass on cut section [1, 2]. Cystic foci are nearly universal and occasionally constitute the dominant feature, prompting radiological and gross pathological diagnoses of multilocular renal cyst [1, 2]. The microscopic appearance of these lesions also mimics cystic nephroma. Within these typically large and relatively homogeneous tumors, discrete foci of necrosis and hemorrhage are common on microscopic analysis [1, 2]. On the contrary, the tumor in our patient appeared as a huge heterogeneous cystic mass on enhanced CT. Gross and microscopic pathological analysis showed that the cysts contained necrotic tissues. These findings are not typical of CCSK.

Radiographically demonstrable calcification is observed in 3–17 % of primary Wilms tumors [3, 4], but calcification is rarely reported in CCSK. Glass et al. reported calcification in CCSK, which was detected as acoustic shadowing on sonography, indicating a calcific deposit [5]. In the present case, calcification was detected on CT. The pathological findings revealed that the calcification was present around the sphacelus, which is considered dystrophic calcification attributable to necrosis [6]. These results suggest that huge areas of necrosis and calcification are not reasons to reject a diagnosis of CCSK.

The abnormal karyotype t(10;17)(q22;p13) has been reported previously in CCSK [7], and CpG sites in the *THBS1* gene were shown to be specifically hypermethylated in CCSK [8]. A new chromosomal abnormality was detected in our patient, 46,XY,der(3)(3pter → 3q23::7?::3?::7p13 → 7pter)der(7)(X?::7p11.2 → 7q22::3?::Xq13 → Xqter)der(X)del(X)(p11.2p22.1)t(X;7)(q11;?). A complex karyotype, containing chromosomal 3, chromosomal 7, and X chromosomal abnormalities, was detected with spectral karyotyping FISH, in which a fragment of chromosome 7 was added to the partially deleted X chromosome. It is unclear whether this abnormality was involved in the development of CCSK, although renal cell sarcoma (RCC) with an Xp11.2 translocation has been reported [9–11]. Calcification and CCSK occurrence, which were two features of our patient, in an older child or young adult are characteristics of RCC with the Xp11.2 translocation [9–11]. Interestingly, both patients with Xp11.2 RCC and our patient display X-chromosome abnormalities. Therefore, it is likely that an X-chromosome aberration produces an atypical pathogenesis in CCSK, including calcification and huge areas of necrosis.

CCSK is considered an unfavorable histological renal tumor by the National Wilms Tumor Study Group (NWTSG) and JWiTS [1, 2, 12–14]. The survival rate for stage 2 CCSK is reported to be 75 % when doxorubicin is added to the therapeutic regimen [1, 2, 13, 14]. However, necrosis is reported to increase the risk of tumor-related mortality in CCSK [13]. Our patient requires careful follow-up.

## Conclusion

The calcification is not a reason to reject a diagnosis of CCSK.

## Consent

Written informed consent was obtained from the parents of the patient for publication of this Case Report and any accompanying images. A copy of the written consent is available for review by the Editor-in-Chief of this journal.

## References

[1] Argani P, Perlman EJ, Breslow NE, Browning N, Green D, D’Angio G (2000). Clear cell sarcoma of the kidney: a review of 351 cases from the National Wilms Tumor Study Group Pathology Center. Am J Surg Pathol.

[2] Gooskens SL, Furtwangler R, Vujanic GM, Dome JS, Graf N, van den Heuvel-Eibrink MM (2012). Clear cell sarcoma of the kidney: a review. Eur J Cancer.

[3] Dickson PV, Sims TL, Streck CJ, McCarville MB, Santana V, McGregor L (2008). Avoiding misdiagnosing neuroblastoma as Wilms tumor. J Pediatr Surg.

[4] Masuda H, Azuma H, Nakajima F, Watsuji T, Katsuoka Y (2004). Adult Wilms’ tumor with calcification untreated for 5 years—a case report. BMC Urol.

[5] Glass R, Davidson A, Fernbach S (1991). Clear cell sarcoma of the kidney: CT, sonographic, and pathologic correlation. Radiology.

[6] Salamat MS (2010). Robbins and Cotran: Pathologic Basis of Disease.

[7] O’Meara E, Stack D, Lee CH, Garvin AJ, Morris T, Argani P (2012). Characterization of the chromosomal translocation t(10;17)(q22;p13) in clear cell sarcoma of kidney. J Pathol.

[8] Ueno H, Okita H, Akimoto S, Kobayasi K, Nakabayashi K, Hata K (2013). DNA methylation profile distinguishes clear cell sarcoma of the kidney from other pediatric renal tumors. PLoS One.

[9] Nagashima Y, Kuroda N, Yao M (2013). Transition of organizational category on renal cancer. Jpn J Clin Oncol.

[10] Kuroda N, Mikami S, Pan CC, Cohen RJ, Hes O, Michal M (2012). Review of renal carcinoma associated with Xp11.2 translocations/TFE3 gene fusions with focus on pathobiological aspect. Histol Histopathol.

[11] Sukov WR, Hodge JC, Lohse CM, Leibovich BC, Thompson RH, Pearce KE (2012). TFE3 rearrangements in adult renal cell carcinoma: clinical and pathologic features with outcome in a large series of consecutively treated patients. Am J Surg Pathol.

[12] Kalapurakal JA, Perlman EJ, Seibel NL, Ritchey M, Dome JS, Grundy PE (2013). Outcomes of patients with revised stage I clear cell sarcoma of kidney treated in National Wilms Tumor Studies 1–5. Int J Radiat Oncol Biol Phys.

[13] Zhuge Y, Cheung MC, Yang R, Perez EA, Koniaris LG, Sola JE (2010). Pediatric non-Wilms renal tumors: subtypes, survival, and prognostic indicators. J Surg Res.

[14] Oue T, Fukuzawa M, Okita H, Mugishima H, Horie H, Hata J (2009). Outcome of pediatric renal tumor treated using the Japan Wilms Tumor Study-1 (JWiTS-1) protocol: a report from the JWiTS group. Pediatr Surg Int.

